# A calix[4]arene-based supramolecular nanoassembly targeting cancer cells and triggering the release of nitric oxide with green light

**DOI:** 10.3762/bjnano.16.75

**Published:** 2025-07-03

**Authors:** Cristina Parisi, Loredana Ferreri, Tassia J Martins, Francesca Laneri, Samantha Sollima, Antonina Azzolina, Antonella Cusimano, Nicola D’Antona, Grazia Maria Letizia Consoli, Salvatore Sortino

**Affiliations:** 1 Department of Drug and Health Sciences, University of Catania, I-95125 Catania, Italy; 2 Istituto di Chimica Biomolecolare, C.N.R., I-95126 Catania, Italy; 3 Institute for Biomedical Research and Innovation, National Research Council (CNR), I-90146 Palermo, Italy

**Keywords:** calixarenes, cell targeting, fluorescence, light, nitric oxide

## Abstract

We have designed and synthesized a novel calix[4]arene derivative bearing four choline appendages as recognition targeting ligands and one amino-nitrobenzofurazan as a fluorescent labelling unit at the opposite sides of the calixarene molecular scaffold. Due to its amphiphilic character, this compound is well soluble in water, forming supramolecular assemblies that are ca. 170 nm in diameter. The nanoassembly selectively targets cancer cells that overexpress the choline transporters, and it can be visualized thanks to the fluorescent tag. The fluorogenic unit also acts as a green light harvesting center, making the nanoassembly a photo-nanoreactor able to encapsulate a hydrophobic nitric oxide (NO) photodonor, otherwise activatable with blue light, and encouraging the NO release with the more biocompatible green light probably by an intra-cage photoinduced electron transfer.

## Introduction

Calix[*n*]arenes are a family of polyphenolic macrocycles, characterized by the presence of a cavity with remarkable hosting properties and synthetic versatility [1–5]. Water soluble calixarenes can be obtained by the introduction of appropriate hydrophilic moieties in the calixarene molecular scaffold, leading to a good biocompatibility and low immunogenicity and paving the way for a variety of applications in the biomedical field [1,6–9]. Due to their amphiphilic character, calix[*n*]arene derivatives can self-assemble in water medium, leading to nanoaggregates exhibiting much better hosting performances than the single monomers [10]. Aggregates of an amphiphilic calix[*n*]arene covalently integrating specific targeting ligands showed improved cell targeting capability [11]. Besides, nanoassemblies of calix[4]arene derivatives proved to be also very suited host supramolecular nanoreactors to amplify the photochemical performances of otherwise poorly active unconventional photoactivatable drug molecules [12–14] as well as efficient cages to inhibit undesired photodegradation of photosensitive conventional drugs [15].

Nitric oxide (NO) is one of the most extensively studied molecules in the fascinating realm of biomedical sciences. This interest is due to its crucial role as a gaseous signaling molecule in the human body [16–21] and its great potential as an unconventional therapeutic to fight important diseases, including cancer, bacterial infections, and cardiovascular and neurodegenerative disorders [22–36]. The strict dependence of the NO effects on site and doses [37] has made NO generation accurately controlled by light stimuli through suitable NO photodonors (NOPDs) a hot topic in the emerging area of photopharmacology [38–39]. Many molecular systems, supramolecular nanoconstructs, and nanomaterials photoreleasing NO have been developed as potential nanomedicines over the last decades [40–50]. In this regard, generating NO with highly biocompatible long wavelength green or red light is highly desirable over blue or even UV light, not only for its intrinsic low toxicity but also for its deeper tissue penetration.

**Scheme 1:**
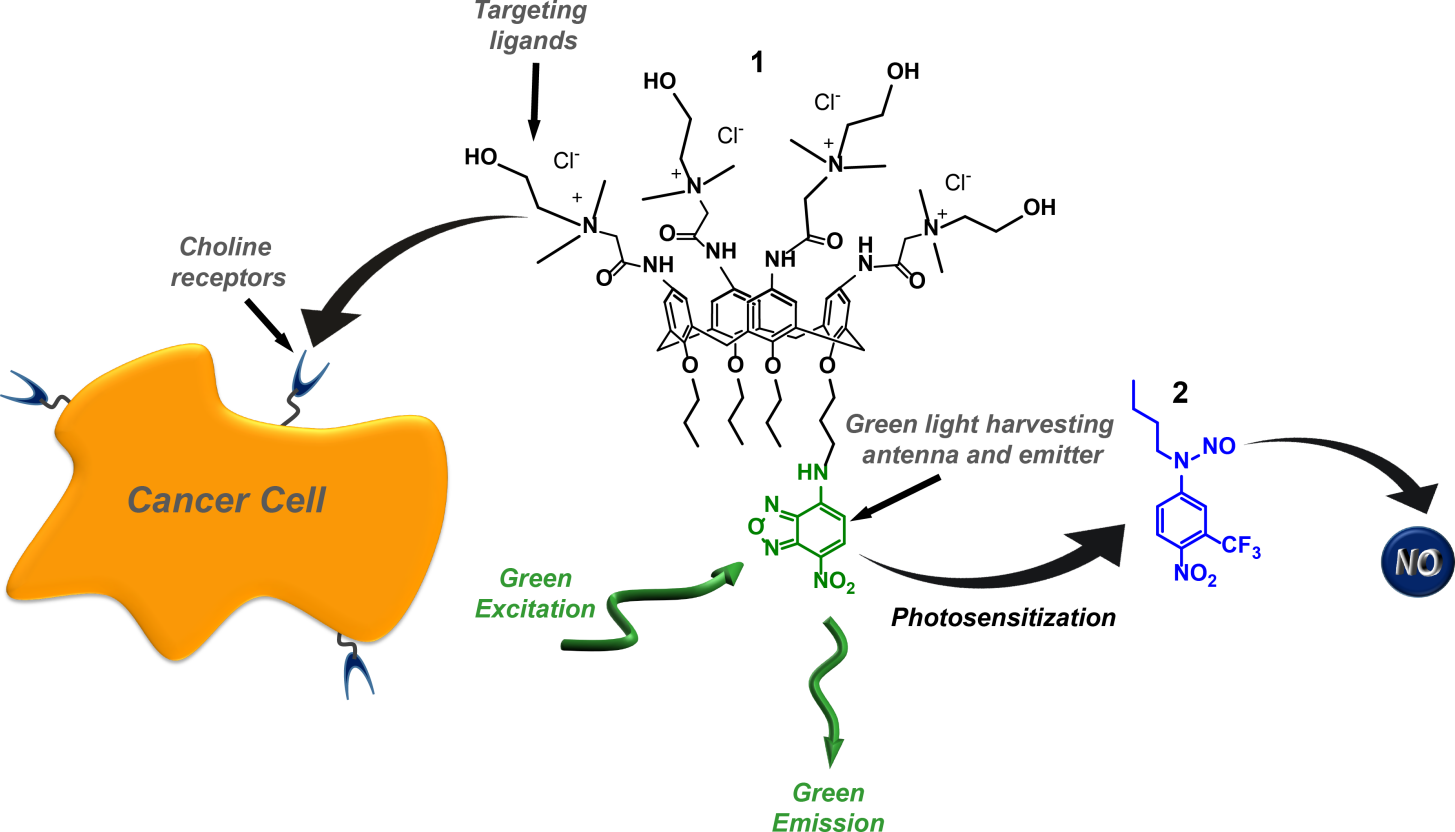
Molecular structures of the multifunctional calix[4]arene **1** and the NOPD **2**.

Our recent work reported a supramolecular approach to trigger the NO release from a blue-light-activatable nitroso-derivative NOPD through red light [51–52]. This was achieved by a bimolecular photochemical reaction between suitable photosensitizers and the NOPD co-encapsulated within different types of biocompatible host systems. Inspired by this work, we thought that a calixarene covalently integrating specific cell-targeting ligands and a suitable chromo-fluorogenic unit can impose the whole structure targeting ability combined with a photoresponsive character. The chromo-fluorogenic component can be exploited for cell tracking and as a suitable low energy light-harvesting antenna to activate photoinduced bimolecular processes with an otherwise blue-light-activatable NOPD encapsulated therein. For this purpose, we report the design and synthesis of the cationic calix[4]arene **1** and its supramolecular nanoassembly with the blue-light-activatable nitroso-derivative NOPD **2** (Scheme 1). We show that (i) **1** self-assembles in water medium into nanoaggregates able to internalize into cancer cells selectively and that (ii) the nanoaggregates of **1** are able to effectively encapsulate the water-insoluble NOPD **2** and trigger the NO release with much more biocompatible green light through a photosensitization process, leading to an improvement of more than 100 nm in terms of excitation wavelength. Nanoassemblies of **1** specifically target cancer cells overexpressing choline transporters and, after encapsulation of the NOPD **2**, stimulate NO release through a green-light-triggered photosensitization process.

## Results and Discussion

### Design and synthesis

Calix[4]arene **1** integrates four choline moieties at the upper rim of the macrocycle scaffold and the 4-amino-7-nitrobenzofurazan (ABF) fluorophore at the lower rim (Scheme 1). The role of these two components in both cases is twofold. The cationic choline groups have been introduced as specific targeting ligands for cancer cells overexpressing the choline transporters [53–54] and to make the final compound amphiphilic, encouraging its aggregation in water medium. The ABF fluorophore is extensively used as fluorogenic labelling unit in biology [55–59]. Besides its role as fluorescent component for cell tracking, it has been selected as green-light-harvesting antenna to trigger the NO release from the hydrophobic NOPD **2**.

**Scheme 2:**
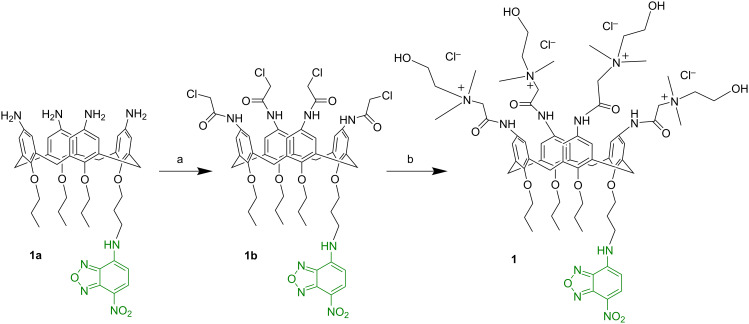
(a) Chloroacetic acid, Et_3_N, CH_2_Cl_2_, r.t., 1 h; (b) *N*,*N*-dimethylethanolamine, THF, reflux, 24 h.

Compound **1** was prepared by a two-step synthesis according to Scheme 2 (see Supporting Information File 1 for details) starting from the known calix[4]arene derivative **1a** [60]. In brief, compound **1a** treated with chloroacetic acid provided compound **1b**, in which four terminal chloromethyl groups are tethered to the calixarene upper rim by amide bonds. The subsequent treatment with *N*,*N*-dimethylethanolamine in THF as a solvent, produced compound **1** bearing choline-like moieties. Compound **1** and its precursor **1b** were characterized by 1D and 2D NMR spectra that confirmed the exhaustive functionalization of the calixarene upper rim (Figures S1–S7, Supporting Information File 1).

**Scheme 3:**
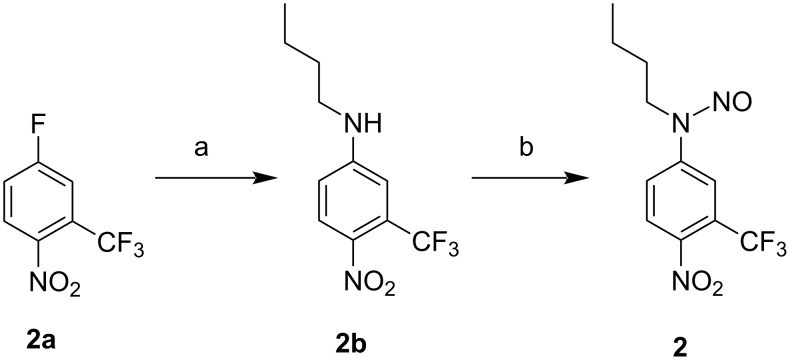
(a) Butylamine, K_2_CO_3_, CH_3_CN, r.t., 24 h; (b) NaNO_2_, THF/CH_3_COOH 2:1, 0 °C → r.t., overnight.

The NOPD **2** contains the same chromophoric motif as our recently reported NOPD [59], differing only by having a C4 alkyl chain instead of a C8. This choice was motivated to encourage a better fit of this NOPD into the calixarene nanocontainer without affecting the already known photochemical NO release properties [61]. Compound **2** was prepared by a two-step synthesis according to Scheme 3 (see Supporting Information File 1 for details). Briefly, the direct coupling of commercially available butylamine with 5-fluoro-2-nitrobenzotrifluoride **2a** in acetonitrile at room temperature gave compound **2b**. Subsequently, nitrosation with sodium nitrite under acid conditions yielded the nitroso derivative **2**. All operations were carried out under a low-intensity level of visible light. Compound **2** and its non-nitrosated precursor **2b** were characterized by 1D NMR (Figures S8,S9, Supporting Information File 1).

### Self-assembling and spectroscopic properties of **1**

**Figure 1:**
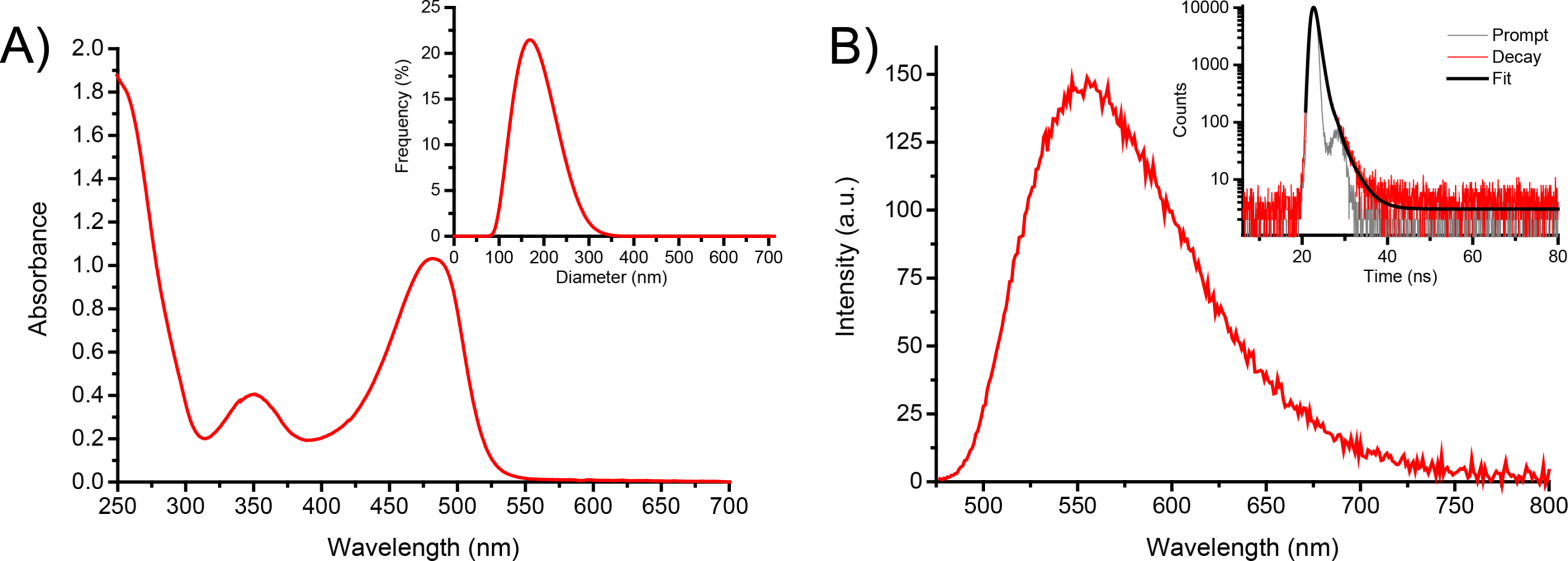
(A) Absorption spectrum of **1** (50 µM) in water. The inset shows the size distribution of the same solution of **1** obtained by DLS. (B) Fluorescence emission spectrum, λ_exc_ = 467 nm, of **1** as in (A). The inset shows the fluorescence decay and the related fitting of the same solutions recorded at λ_exc_ = 455 nm and λ_em_ = 550 nm. *T* = 25 °C.

Due to the polycationic structure, compound **1** showed good water solubility as evidenced by its absorption spectrum dominated by the typical features of the ABF chromophore [55–59,62–64] with a maximum at ca. 480 nm (Figure 1A). However, dynamic light scattering (DLS) measurements evidenced that, in line with its amphiphilic character, compound **1** is not present in the monomeric form but as nanoaggregates ca. 180 nm in diameter with a polydispersity index (PI) of ca*.* 0.4 (inset Figure 1A). This means the sample has a moderate range of particle sizes, however suitable for drug delivery applications.

Figure 1B shows the fluorescence emission spectra of a water solution of **1**. Similar to the absorption, the spectrum is also in line with the typical emission of the ABF fluorophore, with a maximum of ca. 550 nm [62–64]. Despite this spectral similarity, the fluorescence quantum yield of **1** was lower than that reported for the isolated ABF [55–59,62–64] chromophore, being Φ_f_ = 0.02. Besides, the fluorescence lifetime was shorter than that of the ABF chromophore, showing a dominant component (relative amplitude ca. 93%) with τ ca. 0.8 ns (inset Figure 1B). These emission features account for some self-quenching phenomena due to the aggregation of **1**. The aggregates of **1** were quite stable at room temperature for at least 48 h, as evidenced by the unaltered values of the hydrodynamic diameter and the unchanged absorption and emission features over this time window.

### Cytotoxicity and cell targeting properties of **1**

**Figure 2:**
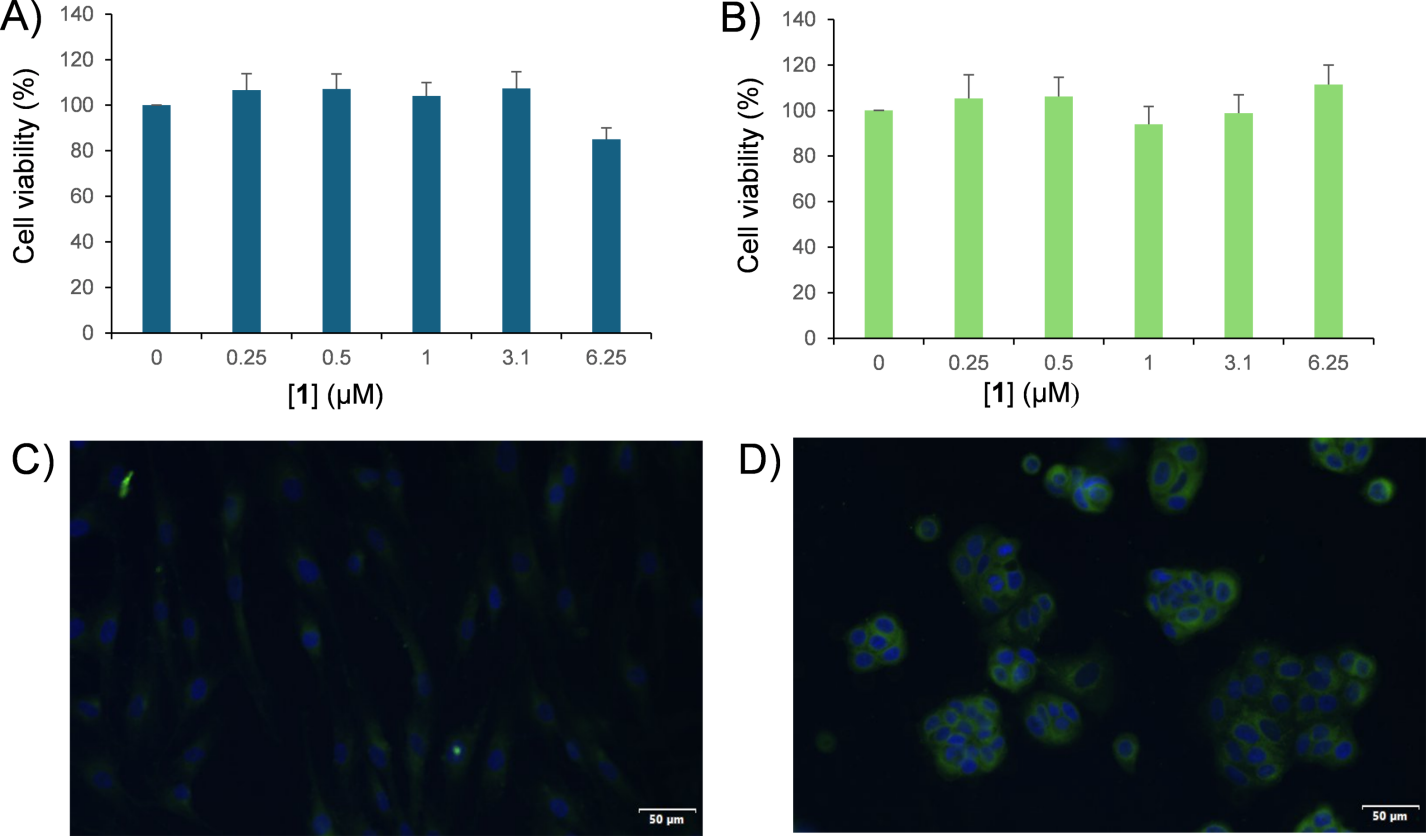
Cell viability effects and cellular uptake of the nanoassembly of **1** (0.5 μM) in HuDe cells (A, C) and MCF7 cells (B, D). Green: nanoassembly of **1**; blue: DAPI stain to visualize nuclei. Scale bar = 50 µm.

The effects of the nanoassembly of **1** on cell viability were evaluated on healthy HuDe cells, a primary dermal human cell line, and on tumor MCF7 cells, a breast adenocarcinoma cell line, by MTS assay. Dose-response experiments were performed. Cells were incubated with increasing concentrations of **1** (0.25, 0.5, 1.0, 3.1, and 6.2 μM) for 24 h. As shown in Figure 2A,B, no cytotoxic effect was observed for any of the tested concentrations up to 6.2 μM.

The four choline ligands of **1** are arranged on the same side with respect to the mean molecular plane. Their cationic nature is expected to reduce their steric hindrance, avoiding self-encapsulation in the calixarene cavity and keeping them inclined to bind specific choline transporter-positive cells overexpressed in tumoral cells [53,65].

To verify if the choline motifs confer to **1** the capability to penetrate selectively choline-positive cells, cellular uptake experiments were performed on tumoral MCF-7 cells and non-malignant dermal HuDe cells, whose different transporter expression level was confirmed by Wester blotting assay (Figure S10, Supporting Information File 1). Based on the cytotoxicity results, the cells were treated with a non-toxic amount of compound **1** (0.5 μM) for 1 h at 37 °C (see Experimental section). As shown in Figure 2C,D, no fluorescence was detected in HuDe cells, while in MCF7 cells we observed an evident, intense diffuse fluorescence at the cytoplasmatic level. The uptake only in tumor cells suggests the nanoassembly of **1** as a potential new agent for selective tumor cell imaging and nanocarrier for tumor cell-targeted drug delivery while sparing normal cells, an essential requirement for a more effective, safe, and precise medicine.

### Host–guest supramolecular complex and NO photorelease

**Figure 3:**
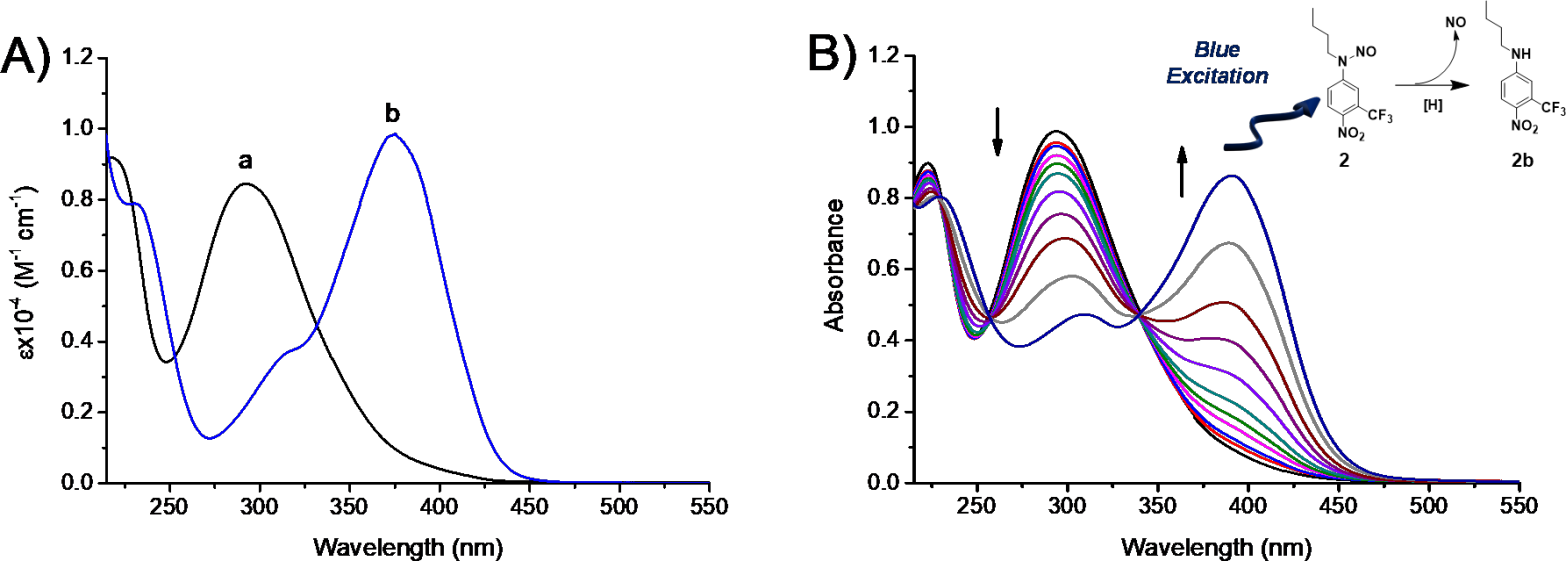
(A) Absorption spectra of NOPD **2** (a) and the non-nitrosated analogue **2b** (b) in water. (B) Absorption spectral changes observed upon exposure of an air-equilibrated NOPD **2** (100 µM) solution at λ_exc_ = 420 nm after 0, 6, 11, 20, 30, 40, 60, 80, 100, 130, and 160 min.

The NOPD **2** is totally insoluble in water. For sake of clarity, Figure 3A shows its absorption spectrum in methanol/water (1:1) and, for comparison, that of its non-nitrosated derivative **2b**. Both compounds exhibit similar molar absorptivity, but the absorption maximum of **2** is by almost 100 nm blue-shifted due to the loss of the push−pull character of the nitroaniline chromophore. Irradiation of **2** with blue light at λ_exc_ = 420 nm leads to the bleaching of the main absorption band at 290 nm and the formation of a new absorption at ca. 400 nm accompanied by the formation of clear isosbestic points (Figure 3B). This photobehaviour accounts well for the loss of NO and the formation of the non-nitrosated **2b** as stable photoproduct (inset Figure 3B), in according to what we have recently reported for an NOPD based on the same chromophoric motif [61]. The quantum yield related to the NO photorelease process was calculated to be Φ_NO_ = 4.5 × 10^−3^, identical to that recently reported for a similar compound bearing a C8 alkyl chain instead of a C4 under the same experimental conditions [61].

**Figure 4:**
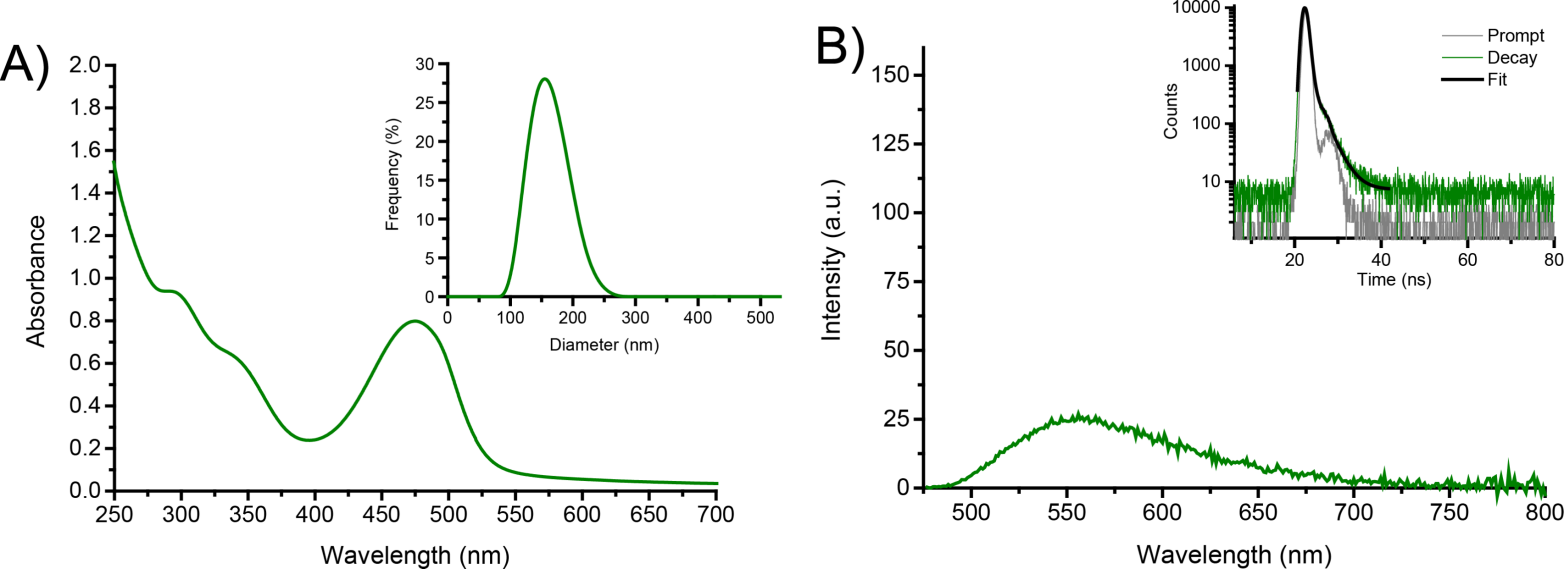
(A) Absorption spectrum of the supramolecular complex **1·2** in water; [**1**] = 50 µM; [**2**] = 40 µM. The inset shows the size distribution of the same solution of **1·2** obtained by DLS. (B) Fluorescence emission spectrum, λ_exc_ = 467 nm, of **1·2** as in (A). The inset shows the fluorescence decay and the related fitting of the same solution recorded at λ_exc_ = 455 nm and λ _em_ = 550 nm. *T* = 25 °C.

The NOPD **2** can be entrapped in the nanoassemblies of **1** in water by a simple and reproducible protocol (see Experimental section) to give a clear colloidal solution. This was confirmed by the appearance of the typical, intense absorption of **2** in the region below 350 nm (Figure 4A). The amount of **2** loaded was ca. 40 µM, corresponding to an encapsulation efficiency of ca. 93%. Note that the encapsulation process does not significantly change the hydrodynamic diameter of the supramolecular nanoassembly, which resulted in ca. 150 nm with a PI of ca. 0.3 (inset Figure 4A). The supramolecular construct **1·2** was stable for days, and its formation and stability can be reasonably attributed to both hydrophobic and stacking interactions between the aliphatic chains and the aromatic regions of the host and guest components. Encapsulation of **2** within the calixarene network did not change the shape and position of the emission spectrum arising from the ABF chromophore (Figure 4B), but significantly reduced the values of the Φ_f_ and τ, being ca. 0.004 and ca. 0.4 ns (relative amplitude ca. 90%) (inset Figure 4B), respectively.

**Figure 5:**
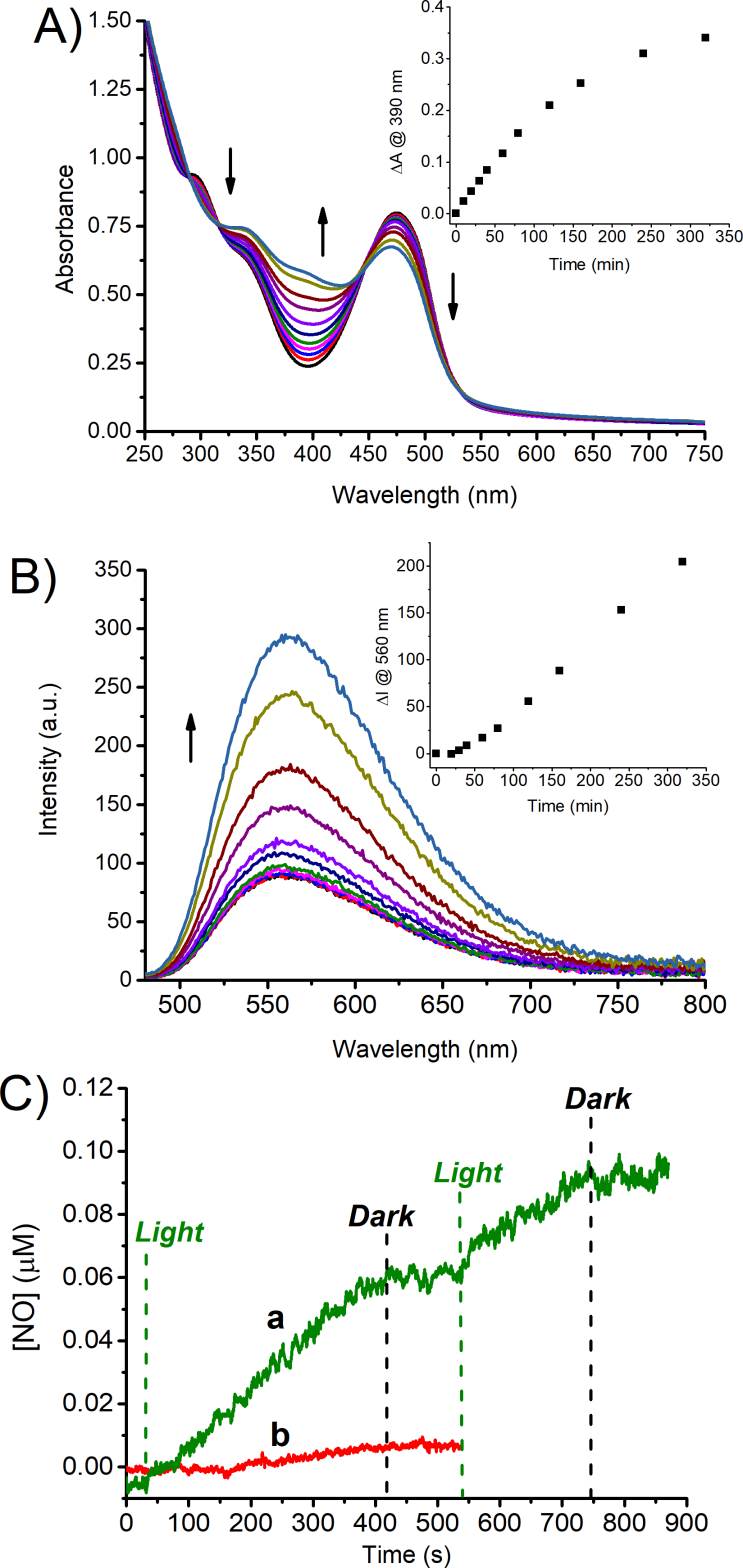
(A) Absorption spectral changes observed upon exposure of a solution of the supramolecular complex **1·2** in water at λ_exc_ = 532 nm for time intervals from 0 to 300 min; [**1**] = 50 µM; [**2**] = 40 µM. The arrows indicate the course of the spectral profile with the illumination time. The inset shows the difference absorbance changes at λ = 390 nm. (B) Evolution of the fluorescence emission spectra corresponding to the sample as in (A) and recorded at λ_exc_ = 467 nm (isosbestic point). The inset shows the fluorescence emission intensity monitored at 560 nm. (C) NO release profiles observed for air-equilibrated solutions of the supramolecular complex **1·2** (a) and the host **1** alone (b) upon alternate cycles of green light irradiation at λ_exc_ = 532 nm. [**1**] = 50 µM; [**2**] = 40 µM. *T* = 25 °C.

The NOPD **2** does not absorb green light (see Figure 3A) and, therefore, is unreactive to this excitation wavelength. However, irradiation of the **1·2** supramolecular complex leads to a photochemical transformation characterized by the increase of the typical absorption of the non-nitrosated derivative **2b** (Figure 5A). In parallel, a restoration of the fluorescence emission reaching a value of Φ_f_ similar to that observed in the absence of **2**, was noted upon photolysis (Figure 5B). These findings account well for the photorelease of NO stimulated by green light. This was unambiguously confirmed by the direct amperometric detection of this radical species through an ultrasensitive NO electrode while alternating cycles of light/dark. Figure 5C shows that the NO generation is achieved exclusively upon green light excitation of the nanoassembly and stops once the irradiation source is switched off. The quantum yield related to the NO photorelease processes was calculated to be Φ_NO_ ~ 4 × 10^−3,^ which is basically the same value observed by direct excitation of **2** with blue light (see above).

Since the ABF chromophore is the only antenna absorbing green light, these results account for a photosensitization process involving this chromophore and the encapsulated **2**. As far as the mechanism of this process is concerned, both singlet–singlet and triplet–triplet energy transfers are ruled out based on the following. Based on the absorption spectra of the ABF and **2**, the lowest singlet state of ABF can be estimated to be several kcal·mol^−1^ lower than that of **2**. This makes, of course, a potential singlet–singlet energy transfer highly endoergonic. In contrast, the triplet state of the ABF chromophore is well known to not be populated unless in cyclohexane [66], making any triplet–triplet energy transfer impossible. According to what was already proposed for the same chromophoric motif of **2** covalently linked to visible light-absorbing antennas [67–69] and other nitroso-derivatives encapsulated within supramolecular reactors [51–52], we believe that the photosensitization process might involve a photoinduced electron transfer. Either reductive or oxidative pathways usually lead to NO loss and the concomitant formation of an anlinyl radical intermediate, which evolves to the stable photoproduct **2b** after H transfer. In our case, such a process involves mainly the lowest singlet state of ABF. This hypothesis is supported well by the reduction of the values of Φ_f_ and τ as a result of the photosensitization process competitive with the fluorescence emission.

## Conclusion

In summary, we have designed and synthesized a multifunctional calix[4]arene, which self-assembles into nanoaggregates in water medium, which are able to selectively internalize in cancer cells due to the specific choline ligands and can be tracked therein thanks to the green light emitter component. This unit also acts as a suitable green-light-harvesting antenna encouraging the NO release from a supramolecularly encapsulated water insoluble NOPD, otherwise activatable by blue light, through a photosensitization process leading to an improvement of more than 100 nm towards biocompatible excitation wavelengths. Studies on the biological effects of the photoreleased NO are currently in progress.

## Experimental

### Chemicals

All chemicals were purchased from Sigma-Aldrich and used as received. Organic solvents were removed under reduced pressure at 35 °C. Synthetic-purity solvents were used. All solvents used for the spectrophotometric studies were spectrophotometric grade.

### Sample preparation

Stock solutions of the NOPD **2** in MeOH were utilized, and the solvent was evaporated under reduced pressure at 35 °C. The resulting film was rehydrated with an aqueous solution of **1** (50 μM) by stirring overnight at room temperature. The final solution was left to equilibrate and filtered. Encapsulation efficiency (EE %) was calculated using the formula


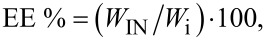


where *W*_IN_ is the amount of guest in the nanoassembly and *W*_i_ is the total amount of guest added initially during preparation.

### Instrumentation

1D and 2D NMR spectra were recorded on Varian UNITY Inova at 500 MHz and Bruker 400^TM^ spectrometers. Chemical shifts (δ) are given in parts per million (ppm), and the coupling constants (*J*) are given in Hz. The following abbreviations are used to designate peak multiplicity: s = singlet, bs = broad singlet, d = doublet, dd = doublet of doublets, t = triplet, q = quartet, quint = quintuplet, and m = multiplet.

Flash column chromatography was performed on silica gel (Merck Kieselgel 60, 230–400 mesh ASTM). The progress of the reactions was followed by thin layer chromatography (TLC) on 5 × 20 cm plates with a layer thickness of 0.2 mm.

UV–vis spectra were recorded with a Jasco V-560 spectrophotometer using quartz cuvettes with an optical path length of 1 cm. Fluorescence emission spectra were recorded with a Spex Fluorolog-2 (mod. F-111) spectrofluorimeter using quartz cuvettes with an optical path length of 1 cm.

Fluorescence lifetimes were recorded with the same fluorimeter equipped with a TCSPC Triple Illuminator. The samples were irradiated by a pulsed diode excitation source Nanoled at 455 nm. The kinetics were monitored at 550 nm, and each solution itself was used to register the prompt at 455 nm. The system allowed for measurements of fluorescence lifetimes from 200 ps. The multiexponential fit of the fluorescence decay was obtained using the following equation:


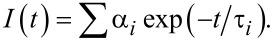


Dynamic light scattering measurements were performed on a ZetaSizer NanoZS90 (Malvern Instrument, UK), equipped with a 633 nm laser, at a scattering angle of 90° and at 25 °C.

In a manner analogous to [70], photolysis experiments were performed by irradiating the samples in solution in a thermostated quartz cell (1 cm pathlength, 3 mL capacity) under gentle stirring, by using a blue-light-emitting diode (λ_exc_ = 415–420 nm) having an irradiance on the samples of ca. 60 mW·cm^−1^ or with a green laser (λ_exc_ = 532 nm, 200 mW). Direct monitoring of NO release in solution was performed by amperometric detection with a World Precision Instrument, ISO-NO meter, equipped with a data acquisition system, and based on direct amperometric detection of NO with a short response time (<5 s) and sensitivity range from 1 nM to 20 mM. The analog signal was digitalized with a four-channel recording system and transferred to a computer. The sensor was accurately calibrated by mixing standard solutions of NaNO_2_ with 0.1 M H_2_SO_4_ and 0.1 M KI according to the reaction:


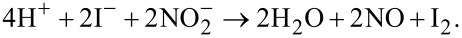


Irradiation was performed in a thermostated quartz cell (1 cm path length, 3 mL capacity, 25 °C) by using the above mentioned green laser (λ_exc_ = 532 nm, 200 mW). NO measurements were carried out under stirring with the electrode positioned outside the light path to avoid NO signal artifacts due to photoelectric interference on the ISO-NO electrode.

### Fluorescence and photodecomposition quantum yields

In a manner analogous to [71], fluorescence quantum yields were determined using optically matched solutions at the excitation wavelength of compounds **1** and its complex with **2**, and fluorescein in acid ethanol (Φ_f_ = 0.78) [72] as a standard through the following equation:


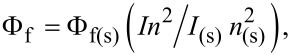


where Φ_f(s)_ is the fluorescence quantum yield of the standard, *I* and *I*_(s)_ are the areas of the fluorescence spectra of the compounds and standard, respectively, and *n* and *n*_(s)_ are the refraction indices of the solvents used for compounds and standard. Absorbance at the excitation wavelength was less than 0.1 in all cases.

Photodecomposition quantum yields Φ_NO_ were determined within the 20% transformation by using the following equation:


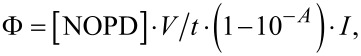


where [NOPD] is the concentration of phototransformed **2**, *V* is the volume of the irradiated sample, *t* is the irradiation time, *A* is the absorbance of the sample at the excitation wavelength, and *I* is the intensity of the excitation light source. The concentration of the phototransformed **2** was determined spectrophotometrically by taking into account the absorption changes at 290 and 400 nm, and Δε_290_ = 8500 M^−1^·cm^−1^ and Δε_400_ = 9600 M^−1^·cm^−1^, respectively. *I* was calculated by potassium ferrioxalate actinometry.

### Cell culture

Primary dermal human cell line HuDe cells (BS PRC 41) were purchased from the Istituto Zooprofilattico Sperimentale of Lombardy and Emilia Romagna (Brescia, Italy) and maintained in culture with DMEM medium (Gibco, Life Technologies), supplemented with heat-inactivated 10% fetal bovine serum (FBS, Gibco, Life Technologies) and 1% antibiotic (penicillin 100 U/mL, streptomycin sulfate 100 mg/mL, Invitrogen). Cells of the human breast adenocarcinoma cell line MCF-7 were obtained from ATCC (HTB-22™, Rockville, MD, USA) and were cultured in Dulbecco’s Modified Eagle Medium (DMEM) supplemented with 10% heat-inactivated FBS (Gibco, Life Technologies), 2 mM ʟ-glutamine, 100 U/mL penicillin, and 100 μg/mL streptomycin. Cells were grown as a monolayer at 37 °C under a controlled humidified atmosphere containing 5% CO_2_.

#### MTS assay

Cell viability assays (MTS) were performed using the CellTiter Aqueous OneSolution kit (Promega, Madison, WI, USA) according to the manufacturer’s protocol. Cells (10 × 10^3^) were seeded into 96-well plates and incubated at 37 °C and 5% CO_2_ for 24 h. Then, cells were treated with increased doses of the nanoassembly of **1**, and after 24 h MTS assays were performed. Cell viability data are expressed as a percentage of the absorbance measured in the control cells, and values expressed as mean ± SD of two separate experiments, each performed in triplicate.

#### Cellular uptake of the nanoassembly of **1**

HuDe cells (1 × 10^5^) and MCF7 cells (2 × 10^5^) were plated in complete medium on coverslips placed in a 12-well plate. After 24 h, cells were incubated in complete medium supplied with **1** (0.5 μM). The incubation was carried out at 37 °C for 1 h. Cells were then washed thrice with PBS, fixed in 4% paraformaldehyde, and processed for immunofluorescence analysis. Images were acquired and collected at 20× magnification using an Olympus fluorescent microscope (Olimpus Evident iX3) at selected channels: GFP channel, emission wavelength 508 nm, excitation wavelength 470 nm and DAPI channel, emission wavelength 455 nm, excitation wavelength 345 nm.

## Supporting Information

File 1 Synthetic procedures and NMR spectra of the synthesized compounds and Western blotting assay.

## Data Availability

Data generated and analyzed during this study is openly available in Zenodo (https://zenodo.org/records/15778109).
