# Supplementary material for: A calix[4]arene-based supramolecular nanoassembly targeting cancer cells and triggering the release of nitric oxide with green light
Source: Beilstein J Nanotechnol. 2025 Jul 3;16:1003–13. doi: 10.3762/bjnano.16.75 (PMC12230331; doi:10.3762/bjnano.16.75)
Supplement: File 1 — Synthetic procedures and NMR spectra of the synthesized compounds and Western blotting assay. [file Beilstein_J_Nanotechnol-16-1003-s001.pdf]

BEILSTEIN JOURNAL OF NANOTECHNOLOGY

Supporting Information

for

A calix[4]arene-based supramolecular nanoassembly targeting cancer cells and triggering the release of nitric oxide with green light

Cristina Parisi, Loredana Ferreri, Tassia J. Martins, Francesca Laneri, Samantha Sollima, Antonina Azzolina, Antonella Cusimano, Nicola D'Antona, Grazia M. L. Consoli and Salvatore Sortino

Beilstein J. Nanotechnol. **2025,** *16,* 1003–1013. doi:10.3762/bjnano.16.75

Synthetic procedures and NMR spectra of the synthesized compounds and Western blotting assay

License and Terms: This is a supporting information file under the terms of the Creative Commons Attribution License (https://creativecommons.org/licenses/by/4.0). Please note that the reuse, redistribution and reproduction in particular requires that the author(s) and source are credited and that individual graphics may be subject to special legal provisions.
The license is subject to the *Beilstein Journal of Nanotechnology* terms and conditions: (https://www.beilstein-journals.org/bjnano/terms)

Synthesis of 5,11,17,23-tetra-amino-25,26,27-tripropoxy-28-propoxy-ABF-calix[4]arene (1a) was performed by a multistep procedure reported in the literature [1], with the variation that the upper rim Boc amino protection was removed using TFA rather than HCl gas.

Synthesis of 5,11,17,23-tetra-amidochloromethyl-25,26,27-tripropoxy-28-propoxy-ABF-calix[4]arene (1b)

Compound **1b** was prepared by adapting a procedure reported in literature [2]. To a solution of compound **1a** (165 mg, 0.20 mmol) in CH_2Cl_2 (2.5 mL), triethylamine (110 µL, 0.76 mmol) and chloroacetic acid (44 µL, 0.76 mmol) were added dropwise. The reaction mixture was stirred at room temperature for 1 h. The organic phase was washed with 0.5 N HCl and water. The organic solvent was removed under vacuum and the crude product was purified by column chromatography on neutral alumina by using ethyl acetate as eluent, and pure compound **1b** was obtained as an orange powder (50 mg, 0.044 mmol, 22 % yield). ^1H NMR (400.13 MHz, $CDCl_3$): δ 0.93 (t, $OCH_2CH_2\mathit{CH_3}$, 3H, J = 7.2 Hz), 0.96 (t, 2 × $OCH_2CH_2\mathit{CH_3}$, 6H, J = 7.2 Hz), 1.86 (m, 2 × $OCH_2\mathit{CH_2}CH_3$, 6H, J = 7.2 Hz), 2.41 (m, $OCH_2\mathit{CH_2}CH_2N$, 2H), 3.17 (d, $Ar\mathit{CH_2}Ar$, 2H, J = 12.8 Hz), 3.19 (d, $Ar\mathit{CH_2}Ar$, 2H, J = 12.8 Hz), 3.64 (m, $OCH_2CH_2\mathit{CH_2}N$, 2H, J = 6.1 Hz), 3.76, 3.80, 3.86 (t, $O\mathit{CH_2}CH_2CH_3$, 2H each), 4.09 and 4.13 (s, CH_2Cl, 4H each), 4.11 (t, $O\mathit{CH_2}CH_2CH_2N$, 2H), 4.36 (d, $Ar\mathit{CH_2}Ar$, 2H, J = 13.2 Hz), 4.41 (d, $Ar\mathit{CH_2}Ar$, 2H, J = 13.2 Hz), 6.14 (d, ArH-NBD, 1H, d, J = 8.4 Hz), 6.29 (m, NH-NBD, 1H), 6.70 and 6.94 (d, ArH, 4H each), 7.99, 8.10, 8.16 (s, ArNH, 2H, 1H, 1H, respectively), 8.49 (d, ArH-NBD, 1H, J = 8.4 Hz). ^{13}C NMR (100.61 MHz, $CDCl_3$): δ 10.4, 23.1, 23.3, 29.2, 31.1, 31.2, 41.3, 42.9, 60.4, 71.5, 98.6, 121.3, 121.7, 124.6, 130.7, 131.2, 134.3, 134.8, 135.3,135.5, 136.2, 143.5, 143.8, 144.3, 153.6, 154.2, 163.9, 164.0.

Synthesis of 5,11,17,23-tetra-amidomethyl-dimethyl-ethanolamine-25,26,27-tripropoxy-28-propoxyABF-calix[4]arene (1)

To a solution of compound **1b** (22.6 mg, 0.0198 mmol) in THF (371 µL), *N,N*-dimethylethanolamine (9.2 µL, 0.092 mmol) dissolved in THF (92 µL) was added. The reaction mixture was refluxed under stirring for 24 hours. After cooling, the precipitate formed was recovered by centrifugation and washed more time with THF (two times) and CH_3CN (4 times). The orange powder was dried under vacuum to give pure compound **1** as an orange powder (29 mg, 0.02 mmol, 98% yield).

^{1}H NMR (400.13 MHz, MeOD): δ 0.97 (t, $OCH_2CH_2\textit{CH}_3$, 3H, J = 7.2 Hz), 1.00 (t, 2 × $OCH_2CH_2\textit{CH}_3$, 6H, J = 7.2 Hz), 1.92 (m, 2 × $OCH_2C\textit{H}_2CH_3$, 4H, J = 7.2 Hz), 1.94 (m, $OCH_2C\textit{H}_2CH_3$, 2H, J = 7.2 Hz), 2.42 (m, $OCH_2C\textit{H}_2CH_2N$, 2H), 3.13 (d, $ArC\textit{H}_2Ar$, 2H, J = 13.3 Hz), 3.15 (d, $ArC\textit{H}_2Ar$, 2H, J = 13.3 Hz,), 3.41 (s, 2 × $N(CH_3)_2$, 12H), 3.43 (s, $N(CH_3)_2$, 6H), 3.45 (s, $N(CH_3)_2$, 6H), 3.71-3.77 (m, overlapped, $NC\textit{H}_2CH_2OH$, $OCH_2CH_2C\textit{H}_2N$, 8H and 2H, respectively), 3.81(t, 2 × $OC\textit{H}_2CH_2CH_3$, 6H, J = 7.24), 3.91 (t, $OC\textit{H}_2CH_2CH_3$, 2H, J = 6.4), 3.98-4.10 (m, $NCH_2C\textit{H}_2OH$, 8H), 4.23 (t, $OC\textit{H}_2CH_2CH_2N$, 2H), 4.32 and 4.36 (s, $NHCOC\textit{H}_2N$, 8H), 4.33 (d, $ArC\textit{H}_2Ar$, 2H, J = 13.2 Hz), 4.46 (d, $ArC\textit{H}_2Ar$, 2H, J = 13.2 Hz), 6.21 (d, ArH-NBD, 1H, J = 7.2 Hz), 6.79 (d, ArH, 2H), 6.82 (d, ArH, 2H), 6.96 (s, ArH, 2H), 7.05 (s, ArH, 2H), 8.48 (d, ArH-ND, 1H, J = 7.2 Hz). ^{13}C NMR (100.61 MHz, MeOD): δ 11.5, 11.7, 25.2, 25.3, 32.9, 33.1, 53.8, 54.7, 57.9, 65.6, 67.2, 68.7, 74.1, 78.9, 79.3, 101.0, 122.9, 124.1, 133.4, 133.7, 133.8, 136.6, 136.8, 137.5, 137.6, 139.2, 146.7, 155.8, 163.8.

Synthesis of *N*-butyl-4-nitro-3-(trifluoromethyl)aniline (2b)

To a solution of butylamine (350 mg, 4.8 mmol) and potassium carbonate K_2CO_3 (663 mg, 4.8 mmol) in acetonitrile (CH_3CN, 10 mL), 5-fluoro-2nitrobenzotrifluoride **2a** (500 mg, 2.4 mmol) was added. The reaction mixture was stirred at room temperature for 24 hours. Then, the solvent was evaporated under reduced pressure and the crude product was purified by flash chromatography eluting with cyclohexane/ethyl acetate (Cy/EtOAc, 9/1 v/v) to give compound **2b** as a yellow solid (428 mg, 68%). ^{1}H NMR (500 MHz, $CDCl_3$): δ 0.98 (t, J = 7.4 Hz, 4H),1.45 (m, J = 14.7, 7.4 Hz, 3H), 1.59 – 1.72 (m, 3H), 3.22 (t, J = 7.2 Hz, 3H), 6.66 (d, J = 9.1, 2.6 Hz, 1H), 6.89 (s, J = 2.7 Hz, 1H), 8.01 (d, J = 9.1 Hz, 1H).

Synthesis of *N*-butyl-*N*-(4-nitro-3-(trifluoromethyl)phenyl)nitrous amide (2)

To a solution of compound **2b** (0.58 mmol, 150 mg) in THF/CH_3COOH (2/1 v/v; 3 mL) cooled at 0 °C with an ice bath, sodium nitrite ($NaNO_2$, 2.32 mmol, 160 mg) was added; the reaction mixture was stirred at 0 °C for 1 h and then at room temperature overnight. The crude mixture was diluted with dichloromethane (DCM, 10 mL) and washed with water (3 × 10 mL), dried over Na_2SO_4 and concentrated to dryness. Purification of the residue by flash chromatography, using Cy/EtOAc (95/5 v/v) as the eluent, gave compound **2** as a pale yellow oil (100 mg, 60 %). ^{1}H NMR (500 MHz, $CDCl_3$): δ 0.96 (t, J = 7.3, 1.5 Hz, 2H) , 1.35 (m, J = 7.4, 1.5 Hz, 1H), 1.53 (m, J = 7.4, 1.4 Hz, 1H), 4.06 (t, J = 7.6, 1.5 Hz, 1H), 7.88 (d, J = 9.1, 1.9 Hz, 1H), 8.13 – 8.08 (m, 1H).

References

1. Lalor, R.; Baillie-Johnson, H.; Redshaw, C.; Matthews, S. E; Mueller, A. *J. Am. Chem. Soc.* **2008,** *130,* 2892. doi:10.1021/ja0782596

2. Rudkevich, D. M.; Verboom, W.; Reinhoudt, D. N. *J. Org. Chem.* **1994,** *59,* 3683. doi:10.1021/jo00092a031

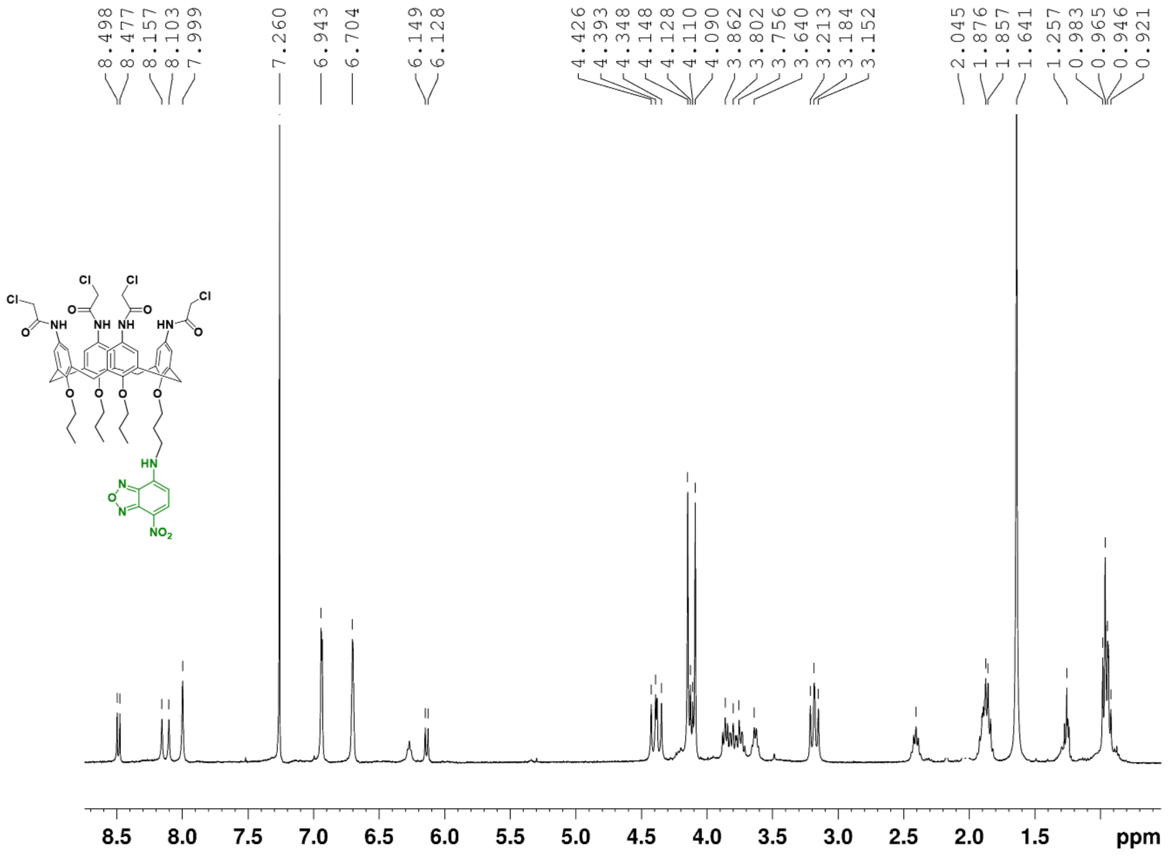

Figure S1: ^{1}H NMR spectrum of compound **1b** (400.13 MHz, 25 °C, $CDCl_3$).

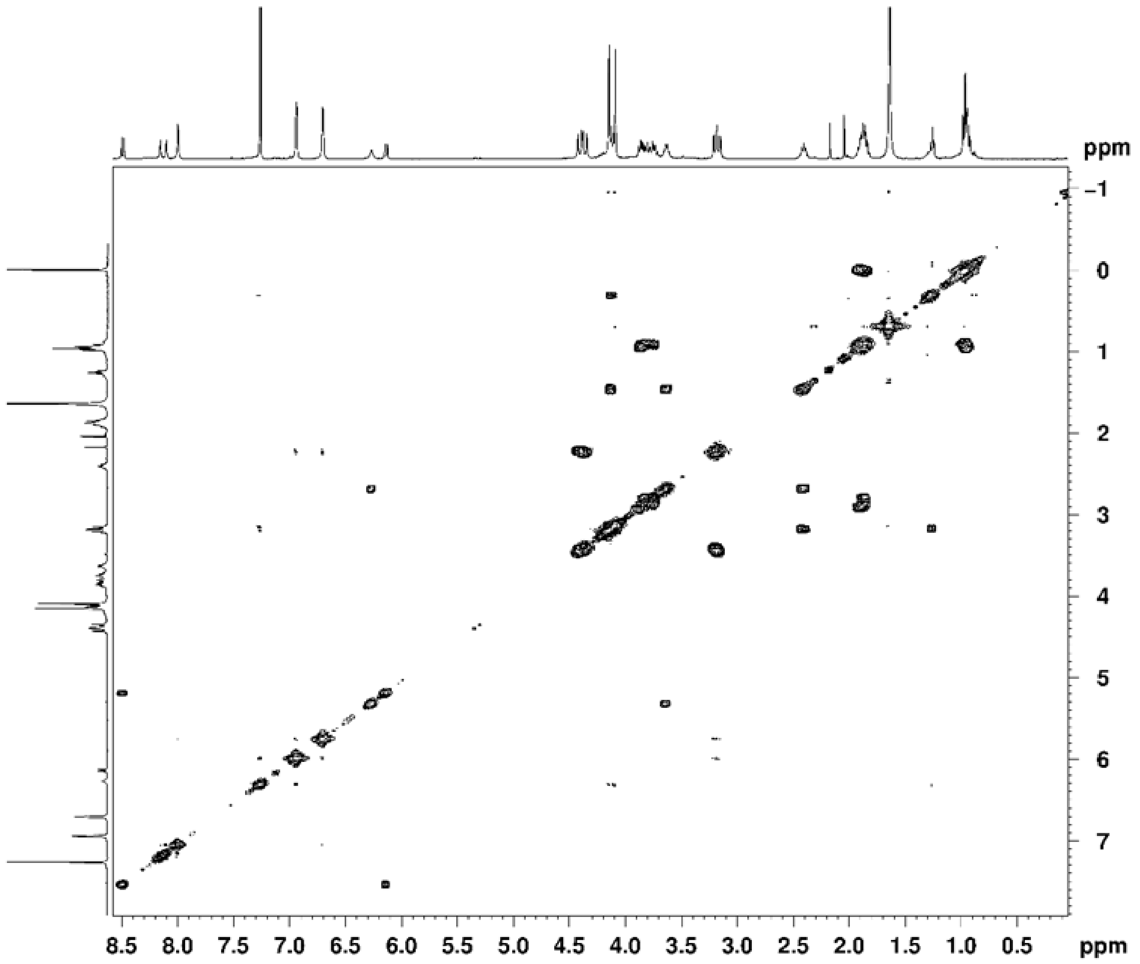

Figure S2: 2D-COSY NMR spectrum of compound **1b** (400.13 MHz, 25 °C, $CDCl_3$).

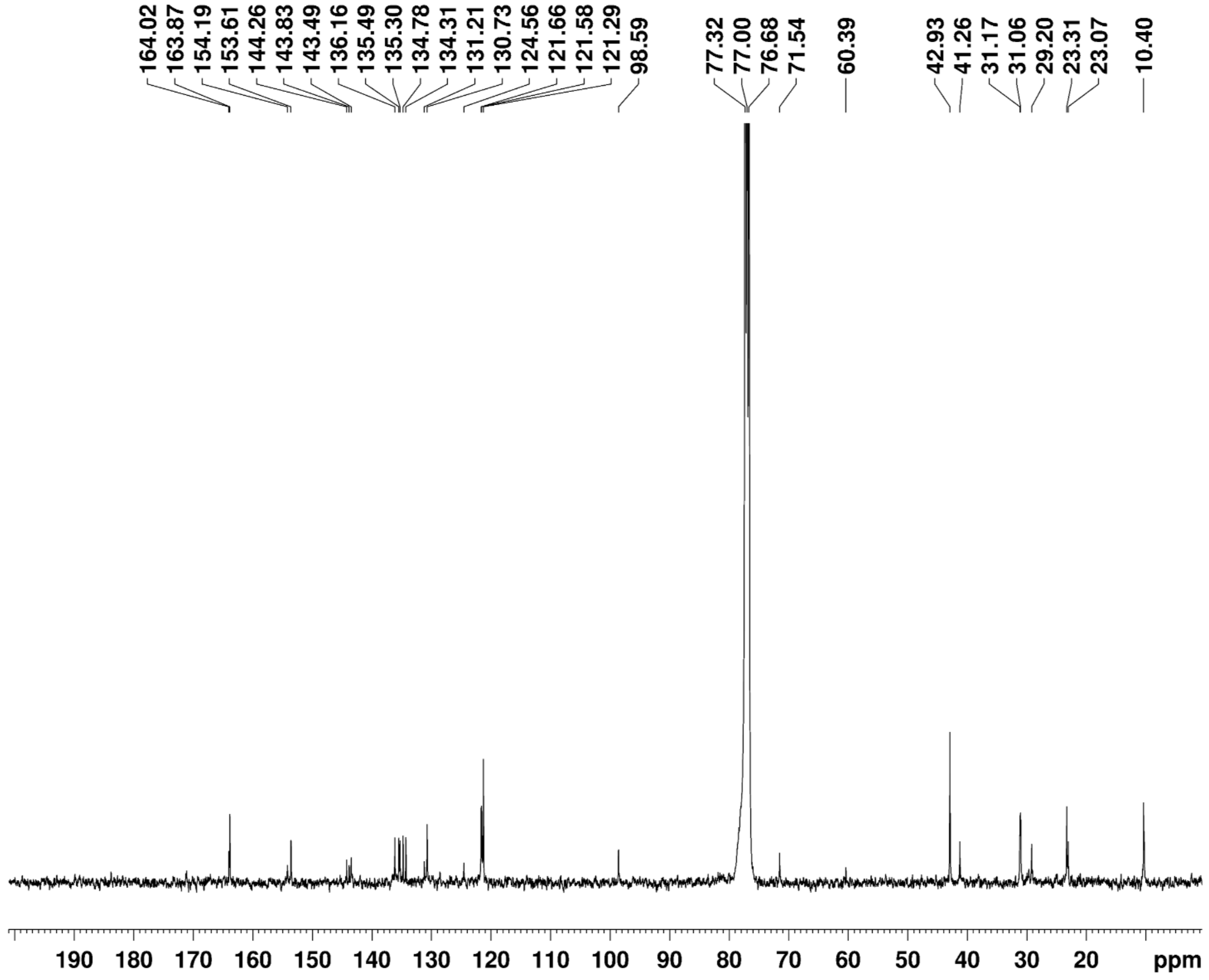

Figure S3: ^{13}C NMR spectrum of compound **1b** (100.61 MHz, 25 °C, $CDCl_3$).

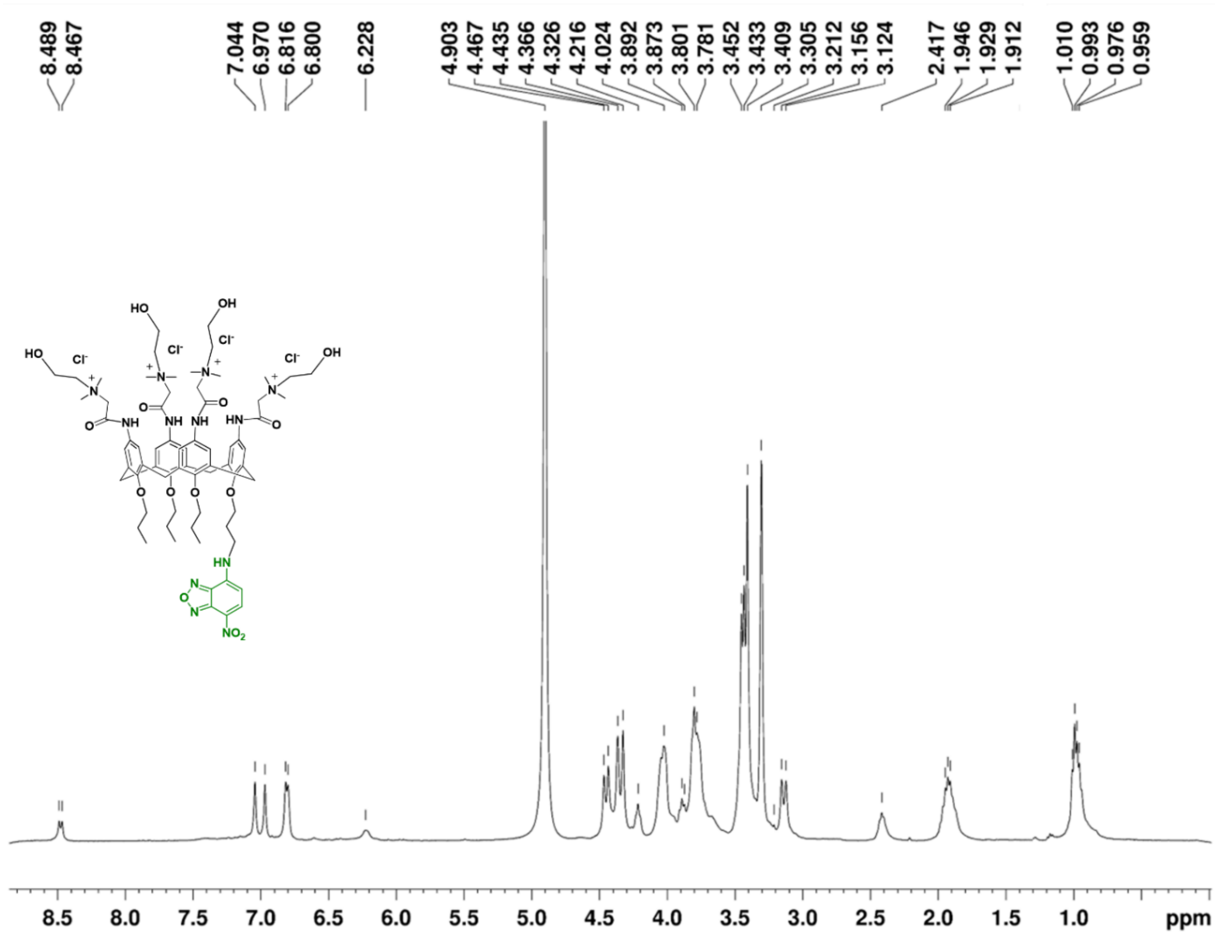

Figure S4: ^{1}H NMR spectrum of compound **1** (400.13 MHz, 25 °C, MeOD).

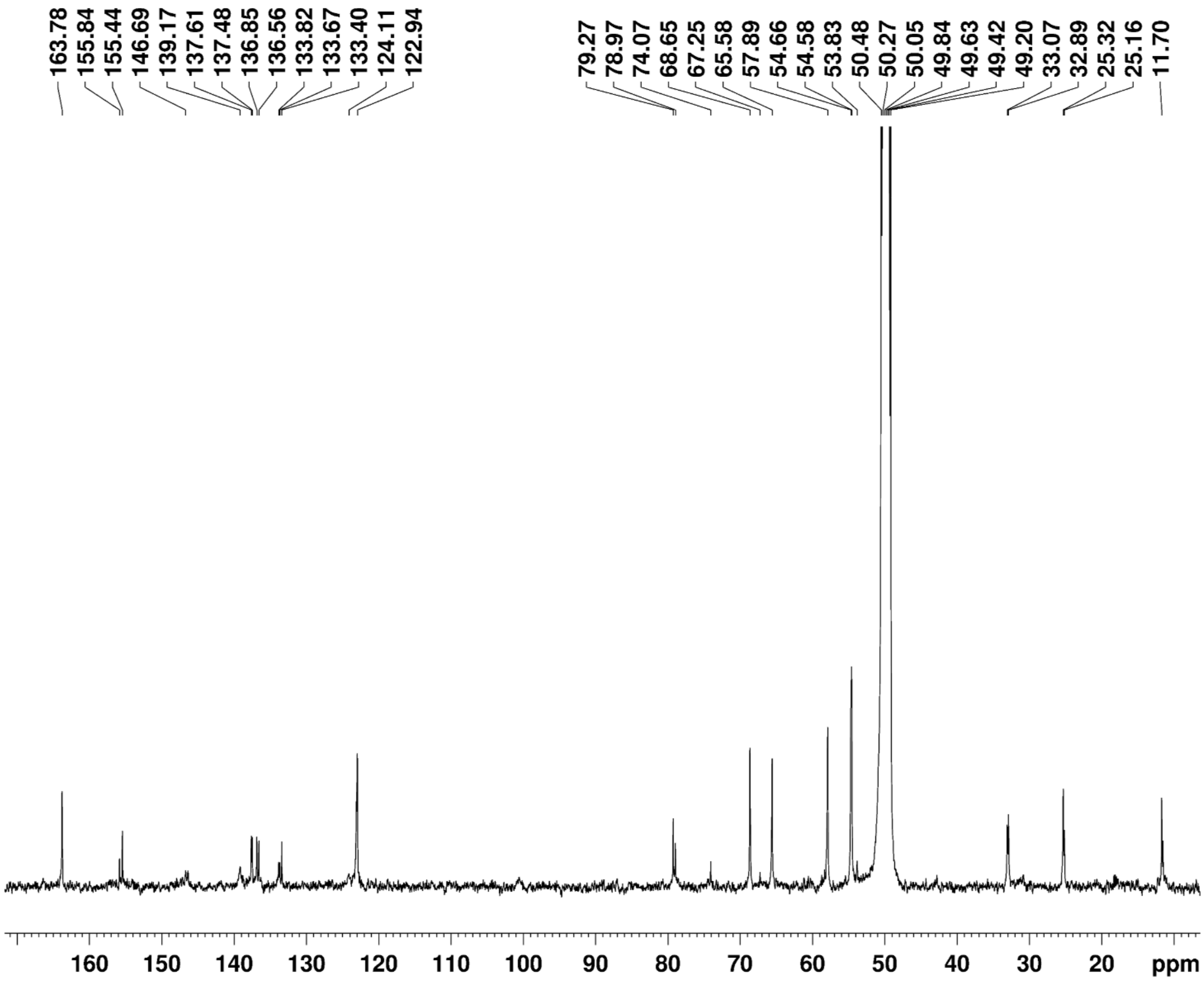

Figure S5: ^{13}C NMR spectrum of compound **1** (100.61 MHz, 25 °C, MeOD).

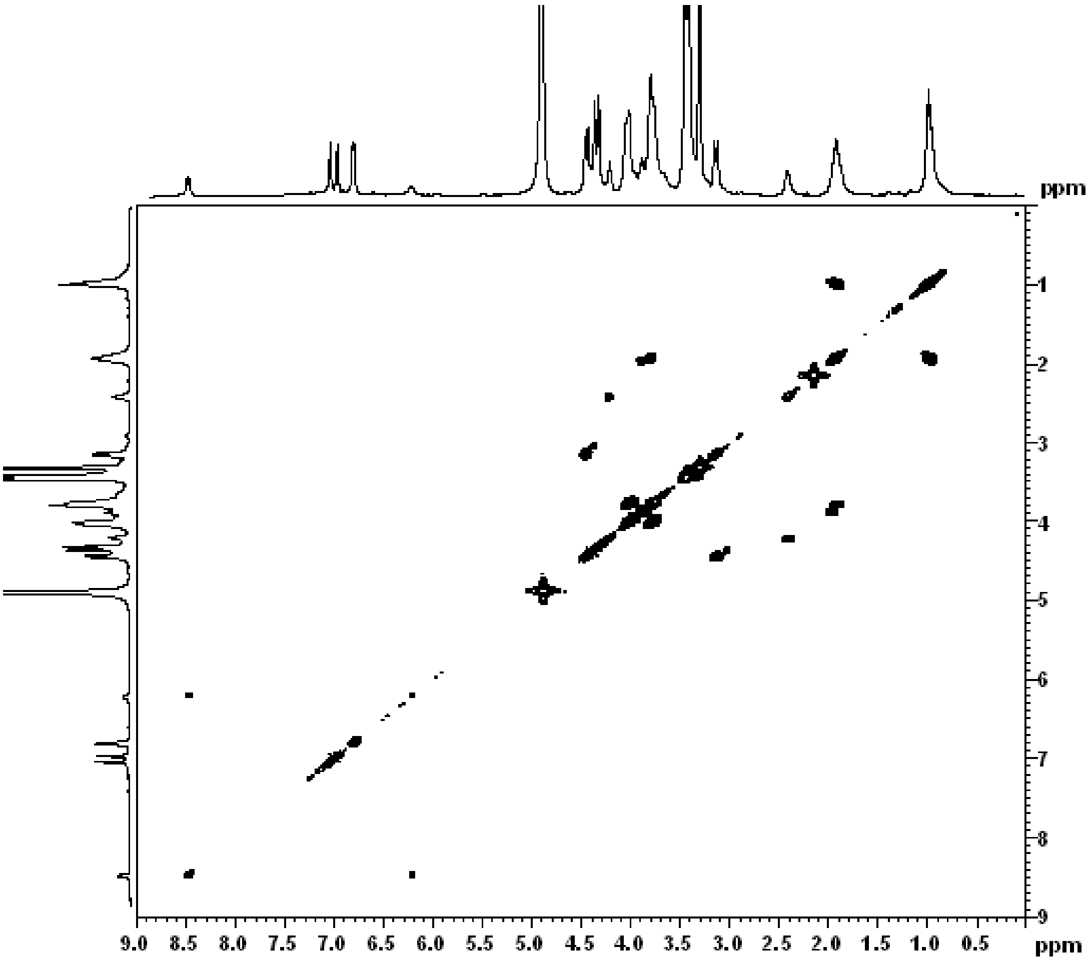

Figure S6: 2D-HSQC NMR spectrum of compound **1** (400.13 MHz, 25 °C, MeOD).

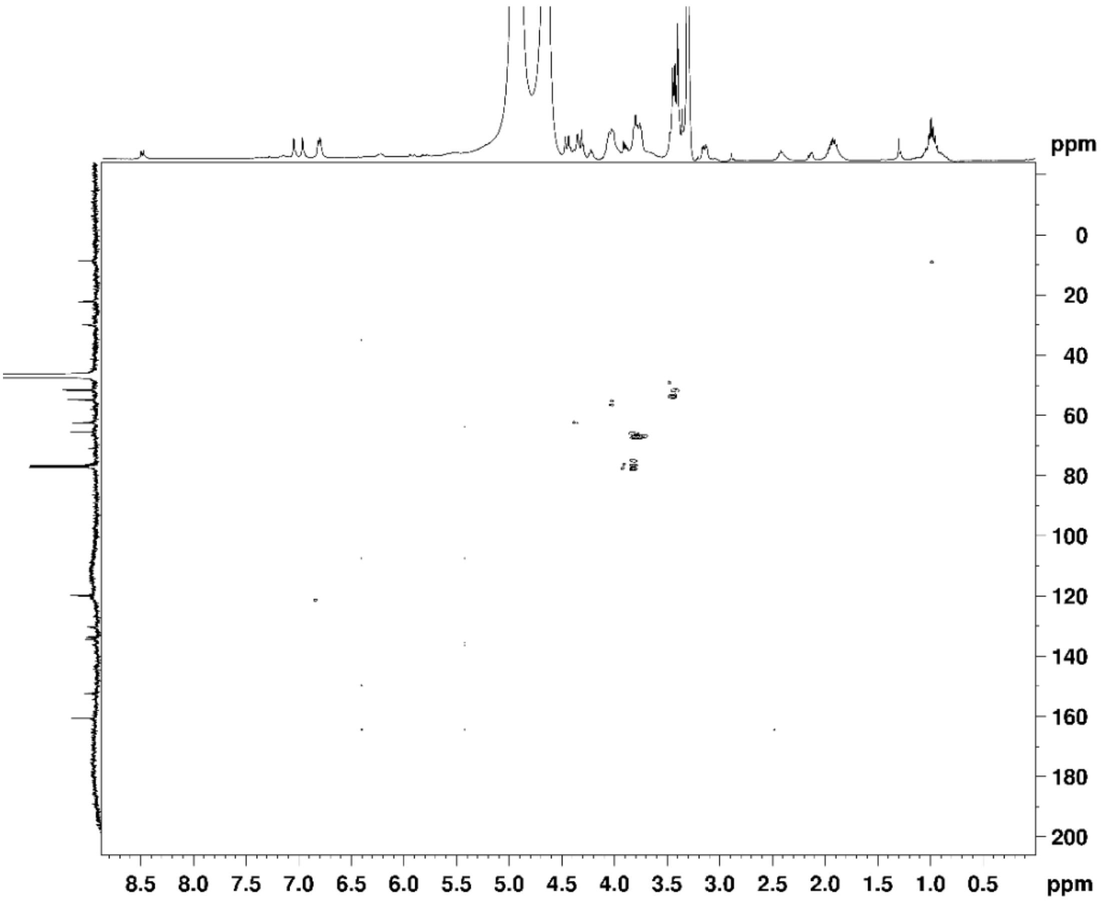

Figure S7: 2D-HSQC NMR spectrum of compound **1** (400.13 MHz, 25 °C, MeOD).

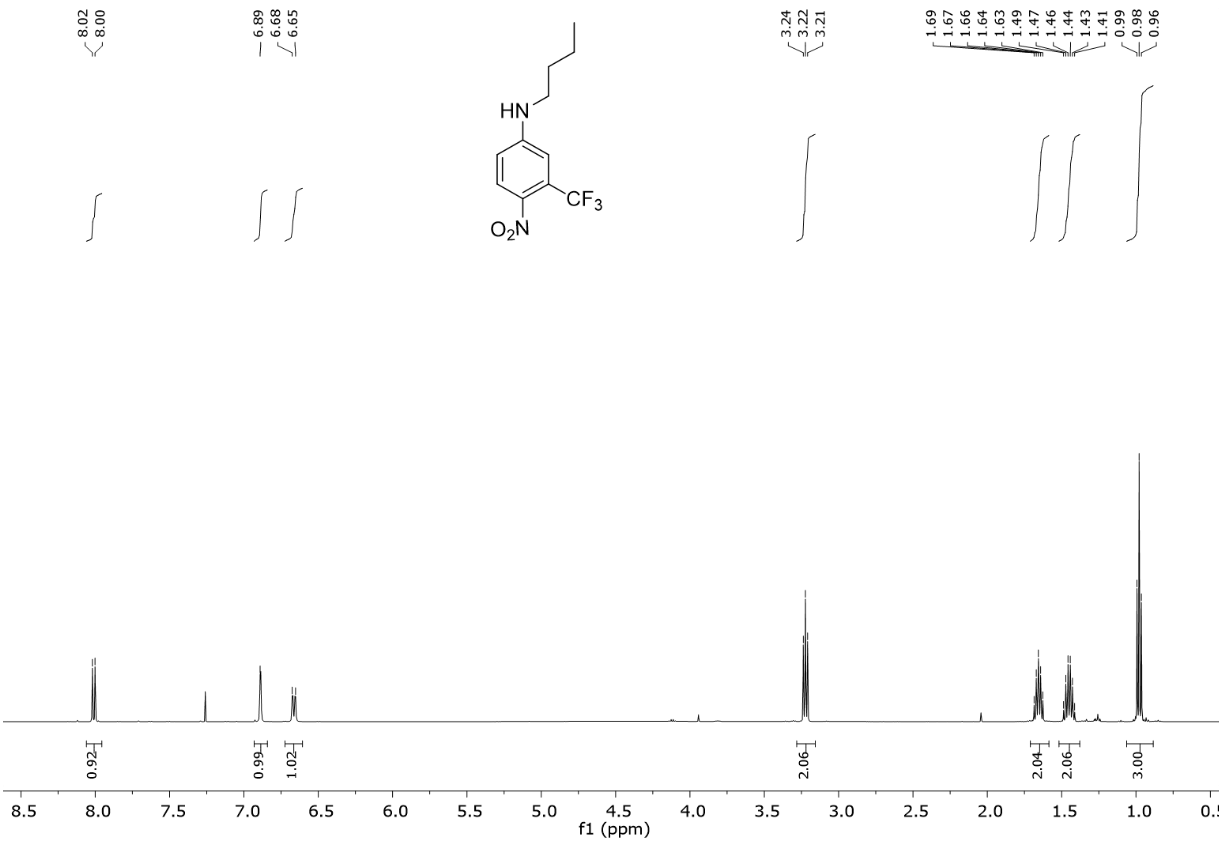

Figure S8: ^{1}H NMR spectrum of compound **2b** (500 MHz, 25 °C, $CDCl_3$).

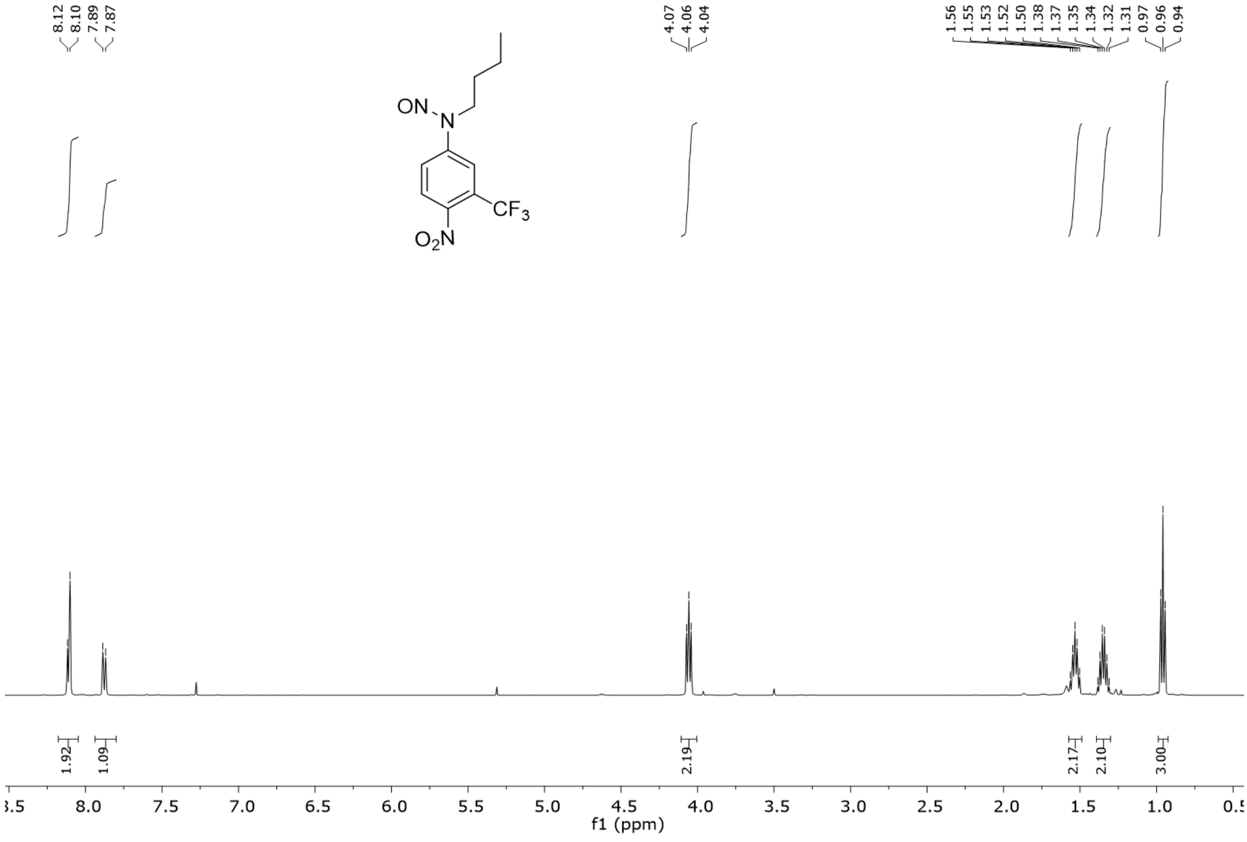

Figure S9: ^{1}H NMR spectrum of compound **2** (500 MHz, 25 °C, $CDCl_3$).

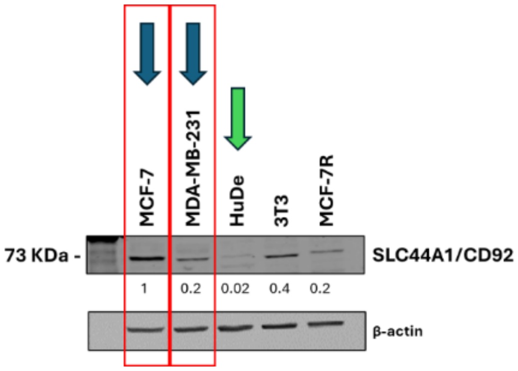

Figure S10: Western blotting for choline receptor expression in breast carcinoma MCF-7 and MDA-MB-231 cells, and normal human dermal fibroblasts (HuDe) cells. SLC44A1 represents just one of the possible choline transporters expressed in cells.
